# A comparison of bolus track and test bolus computed tomography pulmonary angiography and the implications on pulmonary and aortic vessel enhancement, effective dose and suboptimal scan rate

**DOI:** 10.1002/jmrs.724

**Published:** 2023-09-07

**Authors:** Aoife Murphy, Helen White

**Affiliations:** ^1^ Medneo London Centre London UK; ^2^ Birmingham City University Birmingham UK

**Keywords:** bolus track, computed tomography, computed tomography pulmonary angiography, pulmonary embolism, test bolus

## Abstract

**Introduction:**

Bolus track and test bolus are the most commonly used contrast timing protocols to undertake computed tomography pulmonary angiography (CTPA). The aim of this study was to compare test bolus and bolus track contrast enhancement protocols in terms of enhancement of the pulmonary vessels and aorta, radiation dose and suboptimal scan rate to determine the optimal technique for CTPA.

**Methods:**

A total of 200 CTPA examinations (100 using each protocol) performed between January and February 2021 were assessed retrospectively. All scans were performed on a 2x128 Dual Source Siemens Drive Scanner. CT attenuation was measured in Hounsfield Units (HU), with measurements taken from the main pulmonary trunk, right pulmonary artery and left pulmonary artery, ascending and descending aorta. The mean effective dose was calculated from the dose‐length product (DLP). The suboptimal scan rate was calculated as the percentage of examinations below 210HU.

**Results:**

The average HU of the pulmonary arteries was 358 HU ± SD 129.2 in the test bolus group and increased to 394 HU ± SD 133.9 in the bolus track group with a *P* value of ≤0.05. The average HU of the aorta was 235 HU ± SD 82.8 in the test bolus group and increased to 319 HU ± SD 91.8 in the bolus track group with a *P* value of <0.001. Although not statistically significant, the mean effective dose was reduced by 4.2% for the bolus track protocol (2.4 mSv vs. 2.5 mSv, *P* > 0.05). Fewer suboptimal scans were performed with the bolus track protocol (5 scans <210HU Bolus Track vs. 9 scans <210HU Test Bolus).

**Conclusion:**

The bolus track protocol results in increased enhancement of the pulmonary arteries and aorta, with the added benefits of a lower suboptimal scan rate and lower effective dose.

## Graphical abstract

A comparison of test bolus and bolus track contrast enhancement protocols in terms of enhancement of pulmonary vessels and aorta, radiation dose and suboptimal scan rate to determine the optimal technique for computed tomography pulmonary angiography (CTPA). The results of this study justify the use of the bolus track protocol when performing CTPA's with a similar contrast injection rate and scan parameters.

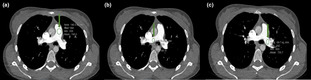

## Introduction

Pulmonary embolism (PE) is a high‐risk mortality disease, with 30% reported mortality when untreated and can cause severe respiratory dysfunction.^1^, ^2^ According to the Centers for Disease Control and Prevention (CDC) PE is the third most common cause of cardiovascular death.^3^ As a result, there is a high diagnostic imaging utilisation to exclude or diagnose this potentially life‐threatening condition. Computed tomography pulmonary angiography (CTPA) is the primary method for diagnosis of PE,^4^ due to its accuracy, speed and 24‐h availability. Confirmation of a PE on a CTPA scan changes patient management as anticoagulation medication can be administered, thereby reducing the risk of death.

A diagnostic CTPA scan depends on sufficient contrast enhancement of the pulmonary arteries with intravenous iodinated contrast to demonstrate the filling defects resulting from the pulmonary emboli. However, the availability of faster scan times due to CT technology advancement has posed a challenge of precisely timing CT data acquisition to coincide with the transit of the contrast through the pulmonary vessels to achieve optimal enhancement. Approximately 5–26% of CTPA scans are non‐diagnostic, with causes including poor contrast enhancement.^5^, ^6^ Poorly enhanced CTPA studies often require repeat scanning with associated radiation exposure, repeat contrast doses and delayed treatment.^7^ The Royal College of Radiologists (RCR) suggest a level of 210 HU as providing acceptable contrast enhancement.^8^ 210 HU is required to identify chronic thrombus from enhancing vessel and acute thrombus has a lower HU than chronic. Therefore, this study recorded the number of examinations below 210 HU in each group, in order to assess the number of suboptimal scans using each technique.

The importance of acquiring a diagnostic CTPA has been further highlighted since the coronavirus disease 2019 (COVID‐19) pandemic. Severe complications of COVID‐19 associated with changes in coagulation put patients at increased risk of PE. A study by Kamenetzky et al.^7^ found 37% of COVID‐19‐positive patients who underwent a CTPA scan were diagnosed with PE. The increased risk of PE due to COVID‐19 resulted in an increase in CTPA requests at the authors' hospital, highlighting the need to identify the optimal CTPA protocol and minimise the number of sub‐optimal CTPA scans.

Multiple CT techniques exist to time contrast enhancement for CTPA acquisition; however, the two most frequently used are bolus tracking and test bolus. The test bolus protocol takes into account the individual patient's physiological parameters such as cardiac rate and breathing rate during the test injection of contrast, as opposed to the bolus tracking method it does not assume the rate of increase in attenuation in the pulmonary artery remains constant.^9^ However, the current literature is not conclusive on the optimal protocol. Henzler et al.^10^ research demonstrated that bolus track resulted in a higher mean enhancement of the pulmonary artery. These findings differ from research by Kamr et al.^11^ who recommend the test bolus protocol and Kerl et al.^12^ who found no significant statistical difference in the enhancement of the pulmonary arteries between protocols. Hence, the aim of this study was to conclude the optimal CTPA protocol and thus provide valuable information for imaging departments when selecting CTPA protocols.

HU measurements were taken from anatomical area's unique to this comparative study. This study included HU measurements taken from the descending aorta in addition to ascending aorta and pulmonary trunk. Sufficient enhancement of the descending aorta allows the rule out of aortic pathologies such as descending thoracic aortic aneurysm, as an alternative cause for similar clinical symptoms. Many studies comparing test bolus and bolus track recorded HU measurements from the pulmonary arteries but not the aorta including Suckling et al.^13^ and Rodrigues et al.^9^ Kamr et al.^11^ analysed area's including the ascending and descending aorta however a test bolus protocol unique to their study was used. HSU et al.,^14^ analysed the main pulmonary artery and descending aorta however they used an empiric timed protocol. In this study, further HU measurements in the distal sections (segmental or subsegmental) of the pulmonary artery vascular tree were not performed, in line with previous research by Goble and Abulkarim^15^ and Suckling et al.^13^ Measurements of small peripheral vessels can lead to potentially inaccurate readings due to partial volume effect from taking a region of interest (ROI) measurement within the small area of peripheral vessels.^9^

The aim of this study was to investigate which CTPA protocol, test bolus or bolus track, resulted in increased enhancement of the pulmonary vessels and aorta and lowest number of suboptimal CTPA scans. Furthermore, as all CT scans pose a radiation risk, the impact of the protocol on radiation dose was also assessed.

## Methods

### Patients

This retrospective study analysed data from 200 adult patient CTPA scans, performed between January and February 2021. Both the bolus track protocol and the test bolus protocol were in use during this time. The decision on which protocol to use was based on the discretion of the radiographer performing the scan. The patient groups were consecutive, unselected patients who were referred for CTPA scans. The bolus track group consisted of 100 consecutive CTPA scans performed using the bolus track CTPA protocol. The test bolus group consisted of 100 consecutive CTPA scans performed using the test bolus CTPA protocol. The CTPA protocol used was identified from data on the Merge (Cambridge, USA) Picture Archive and Communication System (PACS). Exclusions were contrast media site extravasation, severe motion artefact on imaging and multi‐site examinations, for example, CTPA and CT abdomen and pelvis, in order to aid accurate radiation dose calculations. Patient age and gender were collected from Wellbeing Software's (Mansfield, UK) Computerised Radiology Information System (RIS) used at the hospital.

### Imaging technique

Both protocols were performed using the same 2x128 Dual source Siemens Drive Scanner to ensure consistency in measurement and comparability of protocols. Examinations were performed with patients in a supine position and scanned in the caudocranial direction to include the lung bases to apicies. The following scan parameters were used: A collimation/slice thickness of 128 × 0.6 mm; A pitch of 0.8 and gantry rotation time of 0.5 sec; 120 kV and auto mA, with dose modulated using CARE Dose4D. Non‐ionic low‐osmolar intravenous contrast media (CM) Iomeron 400 was injected at 3.5 mL/sec via a 20‐gauge cannula for both the test bolus and bolus track protocol.

The bolus track protocol used Siemens CARE bolus software. A topogram was performed to include the entire chest, followed by a single low dose (100 kV, 23 mAs) axial image to visualise the pulmonary artery. The ROI was placed over the main pulmonary artery. An average of 70 mL of CM was injected via a Bracco power injector, followed by a saline chaser bolus of 10 mL. The trigger threshold inside the ROI was set at 100HU. A 4‐sec interval (scan delay time) was set, during which time the patient was instructed to take a breath in and hold before imaging.

The test bolus protocol used Siemens DynEVA software. Similar to the bolus track protocol, a topogram and control slice was performed. A standard 20 mL test bolus of contrast was injected and sequential low‐dose axial slices at the set ROI in the pulmonary trunk were acquired (100 kV, 23 mAs). The time of peak contrast enhancement was displayed in the form of an ‘attenuation versus time’ graph, which was viewed by the radiographer using the DynEVA software. The time delay from injection to the peak enhancement plus an additional scan delay time of 7 sec was recorded by the radiographer. An average of 74 mL of intravenous iodinated contrast was administered followed by a saline chaser bolus of 10 mL, while the calculated time delay was started and breathing instructions were given.

### Image analysis

**Figure 1:**
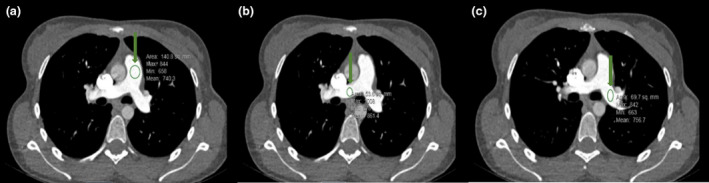
Images demonstrating enhancement of the pulmonary arteries post‐contrast. HU measurements taken in the main pulmonary trunk (A), right pulmonary artery (B) and left pulmonary artery (C).

Images were viewed on PACS for analysis. All images were viewed on an axial reconstruction with 2 mm slice thickness, and angiographic window [window width 870HU, window level 145HU]. Quantitative measurements using hounsfield units (HU) were taken by the lead researcher to analyse contrast enhancement. Measurements were made by placing the ROI cursor in the pulmonary trunk, right pulmonary artery and left pulmonary artery (Fig. 1), ascending and descending aorta. Measurements for the aorta were taken at the level of the main pulmonary artery. The size of each ROI was adjusted to be as large possible within the lumen of the target vessels. The ROI was carefully placed to avoid thrombo‐embolic material, beam hardening artefacts and excluded the vessel wall to avoid atherosclerotic plaques.

### Radiation dose analysis

To estimate the radiation dose for both protocols the dose length product (DLP) was recorded from the RIS and converted to effective dose. The effective dose was calculated as it takes into account both absorbed dose and radiosensitivity of tissue and organs irradiated.^16^ The effective dose was calculated by multiplying the DLP by a tissue weighting factor for the thorax (*E* = DLP *W*_T_ (0.014)). This weighting factor is recommended for use on Siemens CT scan data^17^ and the National Radiological Protection Board^18^ and American Association of Physicists in Medicine.^19^ The effective dose data are presented as the mean effective dose + − 95% confidence interval.

### Statistical analysis

The statistical analysis was performed using the SPSS® software package. Results were considered statistically significant if *P* < 0.05. The Kruskal–Wallis *H* test was used to assess for statistically significant differences between the vessel enhancement, radiation dose, contrast quantity and patient age in both groups. The Chi‐Squared test was used to compare categorical data including gender.

### Ethics

Ethical approval was granted by the Health, Education and Life Sciences (HELS) department at Birmingham City University and Sandwell and West Birmingham NHS Trust Research and Development Department.

## Results

### Patients

There was no statistically significant difference in age between both groups. Both the test bolus and bolus track group had a male majority (Table 1).

**Table 1:** Patient characteristics.

	Test bolus	Bolus track	*P*‐value
Gender	54 male/46 Female	53 male/47 female	0.553 (*P* > 0.05)
Age, years	54 ± SD 17.8	57 ± SD 15.9	0.298 (*P* > 0.05)

### Pulmonary and aortic vessel enhancement

**Figure 2:**
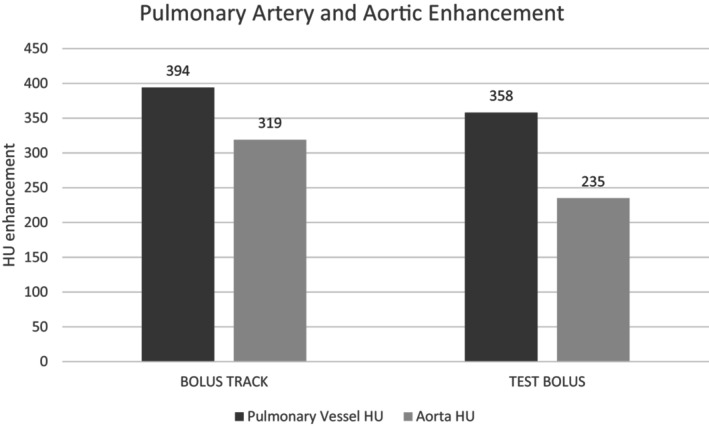
Demonstrated is the increased pulmonary artery average (394 HU vs. 358 HU, *P* = 0.05) and average aortic (319 HU vs. 235 HU, *P* < 0.001) enhancement in the bolus track group.

Pulmonary artery enhancement was higher in the bolus track group, compared to the test bolus group (394 HU vs. 358 HU, *P* ≤ 0.05; Fig. 2). Enhancement of the ascending aorta was significantly higher in the bolus track group, compared to the test bolus group (337 HU vs. 250 HU, *P* < 0.001). Enhancement of the descending aorta was also higher when using the bolus track protocol (301 HU vs. 220 HU, *P* < 0.001; Table 2).

**Table 2:** Vessel enhancement.

Blood vessel	Test bolus mean HU	Bolus track mean HU	*P*‐value
Pulmonary artery average	358 ± SD 129.2	394 ± SD 133.9	0.050 (*P* ≤ 0.05)
Main pulmonary artery	370 ± SD 130.6	404 ± SD 140.6	0.094 (*P* > 0.05)
Right pulmonary artery	351 ± SD 133.7	387 ± SD 133.6	0.040 (*P* < 0.05)
Left pulmonary artery	353 ± SD 125.8	391 ± SD 130.3	0.028 (*P* < 0.05)
Aorta average	235 ± SD 82.8	319 ± SD 91.8	0.000 (*P* < 0.001)
Ascending aorta	250 ± SD 85.7	337 ± SD 92.5	0.000 (*P* < 0.001)
Descending aorta	220 ± SD 82.5	301 ± SD 93.7	0.000 (*P* < 0.001)

### Suboptimal scan rate

Suboptimal enhancement was defined as an examination with mean HU measurements below 210HU, as suggested by RCR for the assessment of the pulmonary arteries.^8^ Nine suboptimal scans were performed in the Test Bolus group and five suboptimal scans were performed in the Bolus Track group, demonstrating an increase of four suboptimal scans in the test bolus group.

### Radiation dose

The mean average effective dose was lower in the bolus track group, in comparison to the test bolus group (2.4 mSv vs. 2.5 mSv, *P* > 0.05). However, this result was not statistically significant.

## Discussion

The bolus track protocol demonstrated increased opacification of the pulmonary arteries and aorta, an increased number of diagnostic CTPA scans and a lower radiation dose when compared to the test bolus protocol. This justifies the use of bolus track protocol at this site when performing CTPA's with similar parameters, also at similar sites with comparable patient population and subset of scanner models.

In this study, the mean pulmonary artery enhancement increased by 10% in the bolus track cohort (394HU vs. 358HU). Several other studies have compared pulmonary enhancement for the test bolus and bolus track protocol, with mixed results. Henzler et al.^10^ found the bolus track protocol (*n* = 30) provided a non‐statistically significant higher mean enhancement in the pulmonary trunk, right and left main pulmonary arteries than the test bolus protocol (*n* = 30).

Kerl et al.^12^ and Johnson et al.^20^ found no significant differences in pulmonary artery enhancement between both techniques. The results from this study are in contrast with Kamr et al.^11^ who performed a large prospective study on 600 patients comparing both techniques. In Kamr et al.^11^ study, the average HU density in the main pulmonary artery was 260.5 HU in the bolus track group and increased to 320 HU in the test bolus group (*P* < 0.002). However, unique to Kamr et al.^11^ study, an extra step was added to the routine bolus track technique. A second ROI cursor was also placed in the ascending aorta and the time difference between the pulmonary artery and aorta was used to calculate the time of peak contrast enhancement. Furthermore, Kamr et al.^11^ used an 18‐gauge cannula with a high flow rate of 5‐6 mL/sec for the test bolus protocol, in comparison to this study where a smaller 20‐gauge cannula with a lower flow rate of 3.5 mL/sec was used.

The different flow rates utilised in both studies offer an explanation for the decreased opacification of vessels in the test bolus cohort of this research. The test bolus theory works on the assumption of a close correlation between the bolus geometry of the test bolus and the main bolus.^21^ However, Platt et al.^22^ stated the test injection was not a useful predictor of time to reach peak enhancement in the aorta when using lower flow rates (3 mL/sec). Platt et al.^22^ reported only when using a faster injection rate of minimum 4 mL/sec, was the test injection delay time significantly correlated (*P* < 0.05) with the main bolus, resulting in greater vascular enhancement. The flow rate used in both cohorts in our study was 3.5 mL per second, which based on Platt's et al.^22^ results would lead to a weak correlation between the test bolus and main bolus of the test bolus protocol. Hence in an effort to improve opacification in the test bolus cohort, the authors' hospital could use a higher flow rate of minimum 4 mL per second.

The aortic enhancement increased substantially in the bolus track cohort, from 250HU to 337HU in the ascending aorta and from 220HU to 301HU in the descending aorta (*P* < 0.001). These results are in agreement with Henzler et al.,^10^ and Kamr et al.,^11^ whose studies showed improved aortic enhancement in the bolus track cohorts. The increase in aortic enhancement is clinically important as the bolus track protocol allowed evaluation of the ascending aorta in an additional 26% of patients and the descending aorta in an additional 38% of patients, assuming a 210 HU threshold. Sufficient aortic enhancement allows the radiologist to rule out aortic pathologies such as aortic aneurysms, as an alternative cause for similar clinical symptoms.^23^

The RCR set an audit target that no more than 11% of CTPA's should have a HU <210 in the main pulmonary artery.^8^ Based on the suboptimal rates in this study, both protocols meet this RCR target and therefore adequate optimal enhancement rates can be achieved with both the bolus tracking and test bolus protocols. However, applying the number of non‐diagnostic scans in this study, using the bolus track protocol only would result in less repeat CTPA examinations. In addition to improving time to diagnosis and patient experience, this would free up the CT scanner to image more patients. Thus, improving efficiency within the CT department and save costs in terms of CT scanner time slots, consumables and salaries.

There was a non‐statistically significant increase of 4.2% in the mean effective dose in the test bolus group (2.5 mSv vs. 2.4 mSv). This was likely due to the test bolus technique requiring more monitoring scans during the test injection, than the bolus track protocol which does not use a test injection. Similarly, Rodrigues et al.^9^ also used 120Kv and reported a 5.9% increase in radiation dose in the test bolus cohort also (4.71 mSv vs. 4.99 mSv). This small decrease in mean effective dose is an important factor when comparing protocols, as per Ionising Radiation (Medical Exposure) Regulations (IR(ME)R)^24^ the radiation dose should be kept as low as reasonably practicable. Radiation risk becomes especially important in young people, particularly females undergoing CTPA examinations as they are more sensitive to ionising radiation due to the amount of breast tissue in the radiation field.^25^

## Limitations

There are limitations to this study. Similar to other retrospective comparative research on this topic,^13^ patient weight was not recorded which could possibly influence whether the differences were due to the protocol used. In addition, the parameters of traditional groups cannot be achieved when imaging a varied population. For example, a large number of patients with a high body weight in the test bolus group could lead to a decreased averaged HU recorded, causing reduced opacification to be linked to the test bolus protocol when in fact the low opacification was due to patient weight. However, this variable is likely non‐threatening to the validity of the HU measurements, as although it was not consistently documented if the contrast volume was altered, radiographers in the study's imaging department routinely compensate for patient weight by increasing or decreasing contrast dose based on patient weight, resulting in consistent HU measurements.

The bolus track protocol used an average volume of 70 mL for the main bolus and the test bolus used an average volume of 74 mL for the main injection bolus. This could result in variations in the opacification depending on the weight of the patients in that group. However, it is likely the 4 mL difference in contrast bolus between both groups was to compensate for weight, to ensure consistent opacification of vessels.

HU measurements were not recorded for distal branches of the pulmonary vessels. Moreover, it is difficult to objectively quantify based on relative variance when scanning has been performed by numerous individuals. While enhancement in the pulmonary trunk and aorta may be optimal, if the distal branches are not sufficiently enhanced the scan could still be less than optimal.^14^ However, since the mean pulmonary arterial (394 HU) and aortic enhancement (319 HU) were both greater in the bolus track protocol, it is inferred the distal pulmonary artery branches were enhanced in a range between those two vessels.^14^

A non‐randomised, single‐centre study design with one model of CT scanner was utilised. Although objective measurements such as HU were used, such methodological design can increase the probability of bias. Furthermore, a single‐centre study design using one CT scanner model can decrease generalisability of results.^26^

## Conclusion

The results of this study suggest the bolus track protocol should be used as the optimal CTPA protocol when using a 20‐gauge cannula with a flow rate of 3.5 mL/sec, as opposed to a test bolus technique. The bolus track protocol had non‐statistically significant improved vascular enhancement of the pulmonary arteries and aorta, which aids PE detection and the rule out of aortic pathologies. The bolus track protocol resulted in less sub‐optimally enhanced CTPA's thus reducing the number of non‐diagnostic studies and therefore unnecessary exposure to radiation and contrast, while enabling faster diagnosis and more efficient use of valuable CT resources. In addition, although non‐statistically significant the bolus track protocol had a lower radiation dose, especially important when imaging radiosensitive breast tissue in young women. These advantages suggest bolus track is a better alternative to test bolus CTPA, when imaging under similar conditions. However, it is recommended that if further comparisons of both protocols are made, that patient weight is recorded to ensure this variable can be accounted for in the analysis and multiple scanner and/or vendor models are used to increase generalisability.

## Conflict of Interest

There are no conflicts of interest.

## Data Availability

The data that support the findings of this study are available on request from the corresponding author. The data are not publicly available due to privacy or ethical restrictions.
